# Sinupret Activates CFTR and TMEM16A-Dependent Transepithelial Chloride Transport and Improves Indicators of Mucociliary Clearance

**DOI:** 10.1371/journal.pone.0104090

**Published:** 2014-08-12

**Authors:** Shaoyan Zhang, Daniel Skinner, Stephen Bradley Hicks, Mark O. Bevensee, Eric J. Sorscher, Ahmed Lazrak, Sadis Matalon, Carmel M. McNicholas, Bradford A. Woodworth

**Affiliations:** 1 Departments of Surgery/Division of Otolaryngology, University of Alabama at Birmingham, Birmingham, Alabama, United States of America; 2 Gregory Fleming James Cystic Fibrosis Research Center, University of Alabama at Birmingham, Birmingham, Alabama, United States of America; 3 Department of Cell, Developmental and Integrative Biology, University of Alabama at Birmingham, Birmingham, Alabama, United States of America; 4 Department of Medicine, University of Alabama at Birmingham, Birmingham, Alabama, United States of America; 5 Department of Anesthesiology, University of Alabama at Birmingham, Birmingham, Alabama, United States of America; Hospital of the University of Pennsylvania, United States of America

## Abstract

**Introduction:**

We have previously demonstrated that Sinupret, an established treatment prescribed widely in Europe for respiratory ailments including rhinosinusitis, promotes transepithelial chloride (Cl^−^) secretion *in vitro* and *in vivo*. The present study was designed to evaluate other indicators of mucociliary clearance (MCC) including ciliary beat frequency (CBF) and airway surface liquid (ASL) depth, but also investigate the mechanisms that underlie activity of this bioflavonoid.

**Methods:**

Primary murine nasal septal epithelial (MNSE) [wild type (WT) and transgenic CFTR^−/−^], human sinonasal epithelial (HSNE), WT CFTR-expressing CFBE and TMEM16A-expressing HEK cultures were utilized for the present experiments. CBF and ASL depth measurements were performed. Mechanisms underlying transepithelial Cl^−^ transport were determined using pharmacologic manipulation in Ussing chambers, Fura-2 intracellular calcium [Ca^2+^]_i_ imaging, cAMP signaling, regulatory domain (R-D) phosphorylation of CFTR, and excised inside out and whole cell patch clamp analysis.

**Results:**

Sinupret-mediated Cl^−^ secretion [ΔI_SC_(µA/cm^2^)] was pronounced in WT MNSE (20.7+/−0.9 vs. 5.6+/−0.9(control), p<0.05), CFTR^−/−^ MNSE (10.1+/−1.0 vs. 0.9+/−0.3(control), p<0.05) and HSNE (20.7+/−0.3 vs. 6.4+/−0.9(control), p<0.05). The formulation activated Ca^2+^ signaling and TMEM16A channels, but also increased CFTR channel open probability (Po) without stimulating PKA-dependent pathways responsible for phosphorylation of the CFTR R-domain and resultant Cl^−^ secretion. Sinupret also enhanced CBF and ASL depth.

**Conclusion:**

Sinupret stimulates CBF, promotes transepithelial Cl^−^ secretion, and increases ASL depth in a manner likely to enhance MCC. Our findings suggest that direct stimulation of CFTR, together with activation of Ca^2+^-dependent TMEM16A secretion account for the majority of anion transport attributable to Sinupret. These studies provide further rationale for using robust Cl^−^ secretagogue based therapies as an emerging treatment modality for common respiratory diseases of MCC including acute and chronic bronchitis and CRS.

## Introduction

Dysfunctional mucociliary clearance (MCC) is a common pathophysiologic process that underlies inflammation and infection in many airway diseases, including chronic rhinosinusitis (CRS) [1], . Transport of the mucus layer within the airway surface liquid (ASL) covering respiratory epithelia is influenced by the transepithelial movement of ions, such as Cl^−^, and the rate at which cilia beat. There is recent growing interest in the use of Cl^−^ secretagogues for treatment of diseases caused by inadequate MCC, including COPD, asthma, and CRS. A primary Cl^−^ channel in respiratory epithelia responsible for proper MCC is the cystic fibrosis transmembrane conductance regulator (CFTR), which is dysfunctional or absent in cystic fibrosis (CF) – resulting in a significant reduction in the rate of MCC. Ongoing drug discovery efforts designed to target CFTR (via high throughput compound library screening) are under development for CF and other lower airway diseases [3]. Enhancing MCC also represents an important therapeutic strategy for patients with active sinus disease (by accelerating clearance of bacterial pathogens), and as a preventative measure among individuals prone to developing recurrent infections (by minimizing the accumulation of inhaled particles, including allergens, pollutants, and debris) [4]–[6]. CRS is a prevalent respiratory illness affecting 16% of the U.S. population and incurs an estimated aggregated cost of 8.6 billion dollars annually in healthcare expenditures [7], [8], [9].

Sinupret is a phytopharmaceutical widely utilized for a variety of respiratory ailments including rhinosinusitis and bronchitis. The compound is a mixture derived from parts of five plants (elder flowers, primrose flowers with calyx, common sorrel herb, European vervain herb, and gentian root), and has been shown efficacious in reducing acute symptoms and signs of sinusitis [10]. The mechanisms underlying therapeutic benefit of the compound are presently unknown, but are thought to be derived, at least in part, from flavonoid components (flavonol glycosides and free aglycones) of the formulation. Numerous additional molecules have been identified in Sinupret (e.g., iridoid glycosides, caffeoyl derivatives, phenolic compounds, triterpenes, plastocyanin, and phenylpropanoids), and many other components have yet to be characterized [11].

Several flavonoids have been demonstrated to strongly activate CFTR-mediated Cl^−^ currents [12]–[14]. Our previous studies have established that Sinupret robustly activates transepithelial Cl^−^ secretion in mice *in vitro* and in airway epithelial cells *in vivo*. The purpose of the present study was to determine whether this bioflavonoid therapeutic activates ciliary beat frequency (CBF) and enhances ASL, and to investigate the underlying mechanism responsible for the Cl^−^ secretagogue activity of Sinupret.

## Materials and Methods

### Primary Cell Culture

Institutional Review Board and Institutional Animal Care and Use Committee approval were obtained prior to initiating these studies. MNSE and HSNE cells were harvested, grown on Costar 6.5-mm diameter permeable filter supports (Corning Life Sciences, Lowell, MA), and submerged in culture medium as previously described [15]–[22]. Media was removed from the monolayers on day 4 after the epithelium reached confluence, and cells fed via the basal chamber. Transgenic CFTR^−/−^ MNSE cultures were also investigated to test CFTR-dependent effects and evaluate contributions from the apical calcium-activated Cl^−^ channel, TMEM16A. Differentiation and ciliogenesis occurred in all cultures within 10 to 14 days. Cultures were used for experiments when fully differentiated with widespread ciliogenesis.

A total of 420 MNSE and 48 HSNE cell monolayers were used to complete these experiments. Cultures with transepithelial resistances (R_t_) >300 Ω*cm^2^ were used to obtain short-circuit current measurements, monitor ciliary beating by inverted microscopy, and measure ASL depth. A minimum of 5 wells were tested per condition.

### Using Chamber Analysis

Transwell inserts (Costar) were mounted in Ussing chambers to investigate pharmacologic manipulation of vectorial ion transport as previously described [15], [17]. Monolayers were evaluated under short circuit current conditions following fluid resistance compensation using automatic voltage clamping (VCC 600; Physiologic Instruments, San Diego, CA). Serosal bath solutions contained (in mM): 120 NaCl, 25 NaHCO_3_, 3.3 KH_2_PO_4_, 0.8 K_2_HPO_4_, 1.2 MgCl_2_, 1.2 CaCl_2_, and 10 glucose. Baths for transwell filters were warmed to 37°C and gassed continuously with a 95%O_2_-5%CO_2_ mixture that provides a pH of 7.4 under conditions studied here. Chemicals were obtained from Sigma (St. Louis, MO) except for Sinupret which was purchased online (Bionorica, Neumarkt, Germany). Ussing chamber experiments were performed with a nominal mucosal Cl^−^ in which 6 mM replaced NaCl in the above solution. The following pharmacologic agents were used: amiloride (100 µM) - blocks epithelial Na^+^ channels, as a means to isolate changes in short-circuit current (ΔI_SC_) secondary to effects on Cl^−^ channel activity [23]; forskolin (20 µM) - activates CFTR by elevating intracellular cAMP, which results in protein kinase A (PKA)-dependent phosphorylation of the CFTR regulatory domain (R-D); tannic acid (100 µM) - an inhibitor of calcium-activated Cl^−^ channels (recently identified as TMEM16A [24]); INH-172 (10 µM)- a CFTR inhibitor that permits determination of CFTR-dependent contributions to I_SC_ [17]; and Sinupret (2.5 mg/ml). Each solution was produced as 1,000× stock and studied at 1× in the Ussing chamber or by CBF analysis. Stock solutions of Sinupret tablets were generated by dissolving in dimethyl sulfoxide (DMSO) followed by removal of insoluble matter by filtration (i.e., concentrations described here do not account for removal of insoluble particulates) [15]. Maximal solubility and activity of the drug was at 2.5 mg/ml with an EC_50_ of 200 µg/ml. DMSO control solutions were tested for comparison in all cases. The I_SC_ was continuously assessed at one current measurement per second. By convention, a positive deflection in I_SC_ under conditions tested here was defined as the net movement of anions from the serosal to mucosal direction.

### Detection of Regulatory Domain (R-D) Phosphorylation and cAMP levels

Cellular cAMP was measured using an ELISA-based detection kit (Cayman Chemicals, Ann Arbor, MI) as previously described [25]. To evaluate R-D phosphorylation, polyclonal NIH-3T3 cells expressing the HA-tagged R-domain were treated with Sinupret solution for 20 minutes, and compared to forskolin (20 uM) ×5 minutes (as a positive control) or DMSO alone (negative vehicle control). Following lysis, equal amounts of protein (50 µg) from total cell lysate were electrophoresed through a 12% sodium dodecyl sulfate-polyacrylamide gel (SDS-PAGE), and immunoblotted with antibody to the HA tag (Covance, Cumberland, VA). Phosphorylation of the R-domain was visualized as a 2–4 kD shift in migration, as previously described [25].

### Fura-2 Imaging

Changes in cytosolic calcium [Ca^2+^]_i_ were measured with dual excitation wavelength fluorescence microscopy after cells were loaded with the Ca^2+^-sensitive dye Fura-2 [26]. HSNE cells were perfused with solutions containing either Sinupret 2.5 mg/ml, DMSO control, or 140 mM UTP (Sigma) as a positive control. Alteration in fluorescence intensity was monitored by a Photon Technology International (Birmingham, NJ) dual wavelength spectrofluorometer (excitation wavelength: 340/380 nm and emission wavelength: 510 nm). The intensity of fluorescence was calculated automatically. The Rmax and Rmin values were determined by the addition of 3 mM Ca^2+^ and 5 mM EGTA plus 5 µM ionomycin, respectively.

### Whole Cell Patch Clamp Analysis

Mouse TMEM16A subcloned in frame within the pEGFP-N1 vector was kindly provided by Professor Lily Y. Jan (University of California, San Francisco). The TMEM16A-GFP construct was as described previously by Schroeder et al. [27] HEK 293 cells were transfected using Lipofectamine 2000 Transfection reagent (Life technologies, Cat. No:11668) according to the manufacturer's instructions. Stably TMEM16A transfected HEK 293 cells were maintained in DMEM supplemented with 10% FBS and 0.4 mg/ml G418 at 37°C in 5% CO_2_ and passaged every 2–5 d. Cells were split and seeded on glass coverslips one day before whole-cell patch clamp analysis. Each cover slip with cells was transferred to an experimental chamber mounted on the stage of an Olympus microscope. TMEM16A currents were recorded in whole cell mode of the patch clamp technique using an Axopatch 2000B amplifier interfaced to a computer through DIGITA 1200 A. The data was recorded and analyzed using Pclamp software (Molecular Devices). During the experiments, cells were perfused with an external solution of the following ionic composition (in mM): 145 CsCl, 2 MgCl_2_, 2 Cacl_2_, 5.5 Glucose, 10 HEPES pH 7.4 (1 N NaOH). The pipette resistance used for whole recording ranged from 3 to 5 GΩ when filled with the following intrapipette solution (in mM): 135 CsCl, 10 KCl, 2 MgCl_2_, 0.1 EGTA, 5.5 Glucose, 10 HEPES, pH 7.2 (1N KOH). All experiments were performed at room temperature. Cells were continuously perfused with the external solution until formation of the recording whole cell configuration. Activators and inhibitors of TMEM16A were applied through the same perfusion system. The rate of perfusion was adjusted to 1 ml/min.

### Single Channel Studies

Conventional inside out patch clamp recordings were performed using CFBE cells expressing WT CFTR. Recording pipettes were constructed from borosilicate glass capillaries (Warner Instruments, Hamden CT) using a Narishige PP83 microelectrode puller and fire polished with a PP90 microforge (Narishige, Tokyo). The pipettes were partially filled with a solution containing (in mmol/l) 150 CsCl, 1 CaCl_2_, 1 MgCl_2_ and 10 HEPES (pH 7.2) and with tip resistances of 6–8 MΩ. Experiments were performed at room temperature (20–22°C). Single channel currents were obtained using an Axopatch 200B patch clamp amplifier (Axon Instruments (AI), Molecular Devices, USA) with voltage commands and data acquisition controlled by Clampex software (pClamp 10, Axon Instruments) and digitized (Digidata 1440A interface, AI) at a sampling frequency of 1 kHz. CFBE cells were seeded on glass coverslips and mounted in a flow through chamber. To obtain seals, bath solutions contained (in mmol/l) 140 NaCl, 4.0 KCl, 1.8 CaCl_2_, 1.0 MgCl_2_, 10 glucose, 10 HEPES, pH 7.4. For inside out patches, bath solution contained (in mmol/l) 150 CsCl, 5 EDTA and 10 HEPES pH 7.4 designed to prevent calcium-activated currents. Sinupret was added to the CsCl bath solution and used at a final concentration of 2.5 mg/ml from a 250 mg/ml stock dissolved in DMSO. Vehicle controls were included in all studies [DMSO (0.01%)]. As an additional control, patches were exposed to ATP (1 mM/PKA 40 units/ml) after Sinupret following perfusion if channel activity was not observed. Confirmation that stimulated currents were due to CFTR was achieved using the inhibitor CFTR(inh)-172 at 10 µM. Single channel recordings were analyzed using pClamp 10 software (AI).

### Ciliary Beat Frequency Analysis

Images were visualized using a 20× objective on an inverted scope (Fisher Scientific, Pittsburgh, Pa.). Data were captured using a Model A602f-2 Basler area scan high-speed monochromatic digital video camera (Basler AG, Ahrensburg, Germany) at a sampling rate of 100 frames per second. Image analysis was performed with the Sisson-Ammons Video Analysis (SAVA) system, version 2.1.6 [28]. For each experiment, a large area of beating cilia within air-liquid interface cultures was identified with the inverted microscope. Images were analyzed with virtual instrumentation software designed specifically for measuring CBF. A baseline recording of CBF was conducted for each cell monolayer prior to applying an apical test solution. All experiments were compared to the corresponding control using vehicle alone (apical fluid addition, by itself, can increase CBF). Each recording was normalized to fold-change over baseline (Hertz pre- to post-treatment). All studies were performed at ambient temperature (23°C).

### Airway Surface Liquid Depth

MNSE cells were washed 3 times with PBS, and then exposed to 100 µM CellTracker Green BODIPY (Invitrogen, Cat#: C2102) in differentiation medium from the basolateral side, and 10 mg/ml Texas Red (Invitrogen, Cat #: D-1863) in FC-70 (Flourinert FC-70, Fisher, Cat #: NC 9062226) from the apical side. Sinupret (2.5 mg/ml) or the vehicle (DMSO) control was added in a 30 µl volume to the apical and basolateral compartments for 30 minutes. Imaging was performed with a Zeiss LSM 710 Confocal Microscope with a Zeiss Plan Apo 20×.8 na dry Objective in 1 micron steps. ASL depth was measured in the orthogonal (head on X-Z) image view.

### Statistical analysis

Statistical analyses were performed using unpaired t tests or analysis of variance with Tukey-Kramer post hoc analysis as appropriate +/− standard deviation. A p value<0.05 was considered statistically significant.

## Results

### Sinupret increases transepithelial Cl^−^ transport through CFTR and calcium-activated pathways in Ussing chamber analysis

**Figure 1:**
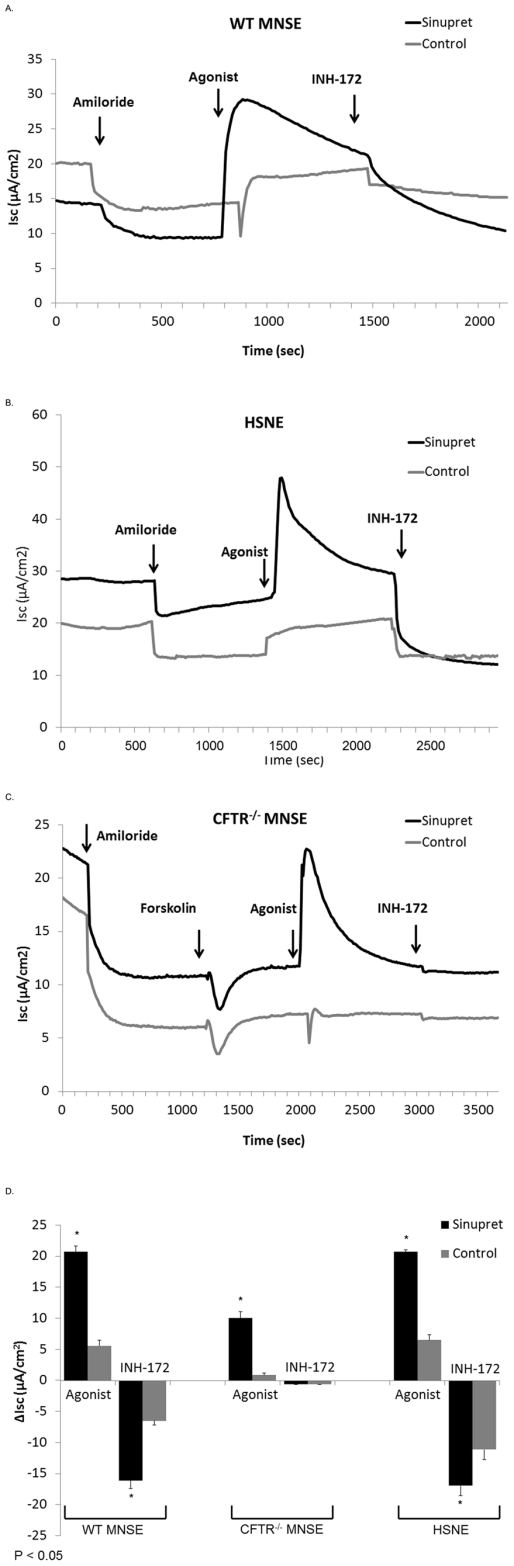
Sinupret is a robust activator of CFTR-mediated and calcium-activated Cl^-^ secretion. Representative Ussing chamber tracings demonstrate pharmacologic manipulation of ion transport with activation of Cl^−^ secretion (Agonist = Sinupret or DMSO control vehicle) in murine nasal septal epithelial (MNSE, wild type and CFTR^−/−^) and human sinonasal epithelial (HSNE) cell cultures following blockade of epithelial sodium channels by amiloride. Strong inhibition of short-circuit current (I_SC_) by the specific CFTR inhibitor INH-172 indicates activation of CFTR-mediated pathways in WT MNSE (A) and HSNE (B). CFTR^−/−^ MNSE tested with forskolin confirm absence of cAMP-dependent CFTR-dependent transport, but retained strong stimulation of anion secretion by Sinupret (C). Summary data for Sinupret-stimulated ΔI_SC_ in WT MNSE, CFTR^−/−^ MNSE and HSNE (D). Sinupret significantly increased ΔI_SC_ (in µA/cm^2^)when compared to controls in both WT (20.7+/−0.9 vs. 5.6+/−0.9, p<0.05) and CFTR^−/−^ MNSE (ΔI_SC_ in µA/cm^2^, 10.1+/−1.0 vs. 0.9+/−0.3, p<0.05). HSNE cultures also exhibited robust Cl^−^ secretion when compared to controls (ΔI_SC_, 20.7+/−0.3 vs. 6.4+/−0.9, p<0.05). There were significant decreases in I_SC_ when cultures were treated with CFTR inhibitor INH-172 in WT MNSE (−16.1+/−1.3 vs. −6.5+/−0.7, p<0.05) and HSNE cells (−17.0+/−1.6 vs. −11.1+/−1.7, p<0.05).

One of the goals of this study was to test whether Sinupret stimulated CFTR or calcium-activated Cl^−^ (TMEM16A) channels on the apical cell surface. The primary method to evaluate anion transport involved pharmacological manipulation of cell cultures mounted in Ussing chambers followed by measurements of short-circuit current (ΔI_SC_). After blocking epithelial sodium channels with amiloride, Sinupret-dependent ΔI_SC_ (in µA/cm^2^) was tested in wild type (WT) MNSE (Figure 1A), CFTR^−/−^ MNSE (Figure 1B), and HSNE cultures (Figure 1C). Summary data is presented in Figure 1D. Sinupret significantly increased ΔI_SC_ when compared to vehicle control in WT MNSE (20.7+/−0.9 vs. 5.6+/−0.9, p<0.05). In addition, Sinupret significantly stimulated Cl^−^ secretion in HSNE cultures when compared to DMSO control (ΔI_SC_, 20.7+/−0.3 vs. 6.4+/−0.9, p<0.05) establishing that the therapeutic is a strong Cl^−^ secretagogue in human sinus and nasal epithelium. The formulation also amplified ΔI_SC_ in CFTR^−/−^ MNSE cultures (ΔI_SC_, 10.1+/−1.0 vs. 0.9+/−0.3, p<0.05), albeit at levels approximately 50% of WT. These data suggest stimulation of transepithelial Cl^−^ secretion is partially mediated through channels other than CFTR.

**Figure 2:**
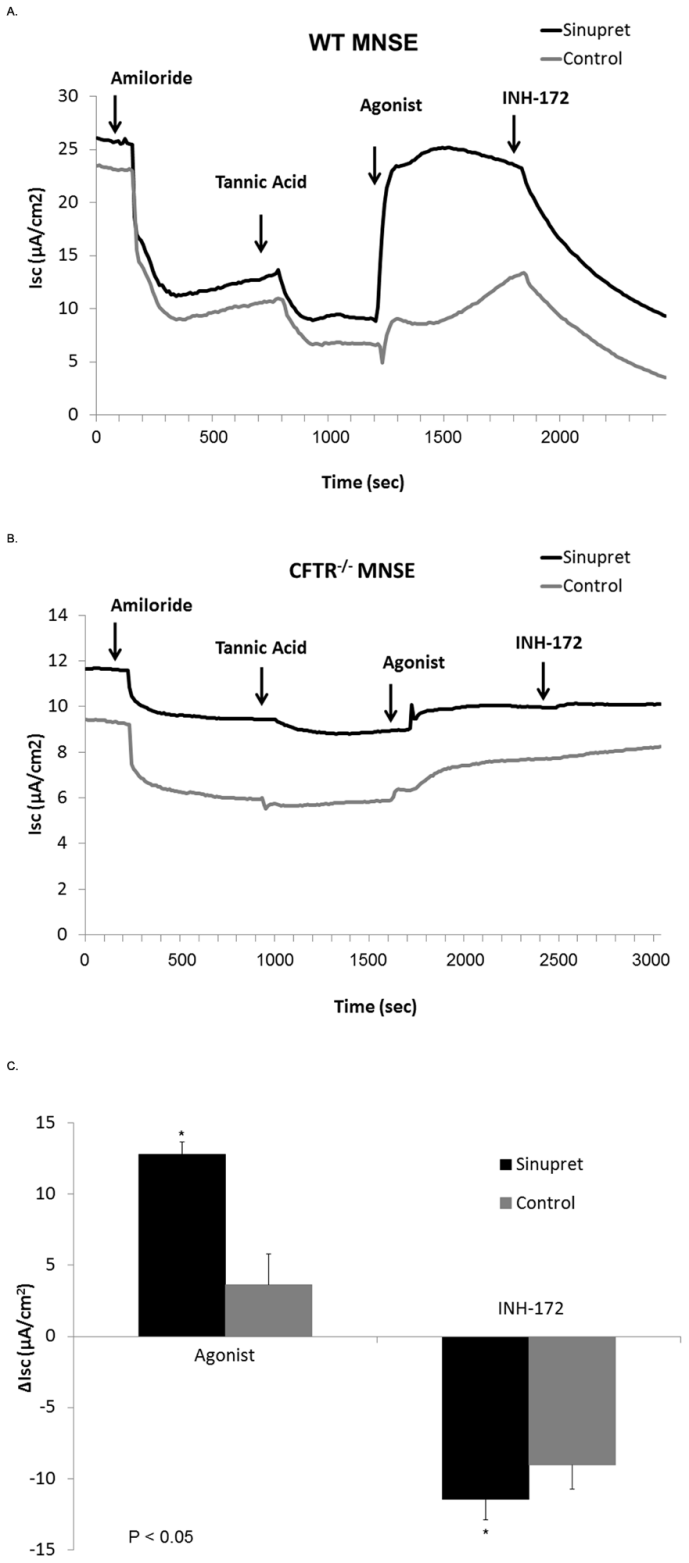
Elimination of TMEM16A-mediated I_SC_ with tannic acid suggests Sinupret stimulates CFTR-mediated Cl^−^ transport. Representative Ussing chamber tracings demonstrate administration of the TMEM16A blocker tannic acid abolishes agonist (Sinupret)-stimulated Cl^−^ secretion in CFTR^−/−^ cells (A). Pre-treatment of WT MNSE shows persistent activation despite elimination of TMEM16A contributions to the I_SC_ (B). Summary data (C) for Sinupret-stimulated ΔI_SC_ in WT MNSE [µA/cm^2^, 12.8+/−2.2 vs. 3.6+/−0.9 (control), p<0.05] following tannic acid block of TMEM16A-mediated I_SC_) and CFTR blockade with INH-172 (−11.5+/−1.7 vs. −9.0+/−1.5, p<0.05).

Another means to investigate overall contribution of CFTR channel activation to the Sinupret -mediated ΔI_SC_ is by blocking CFTR with the specific inhibitor INH-172 or pre-treating the cells with tannic acid (to eliminate calcium-activated I_SC_). We observed significant decreases in I_SC_ when Sinupret-dependent cultures were administered INH-172 in WT MNSE (−16.1+/−1.3 vs. −6.5+/−0.7, p<0.05) and HSNE cells (−17.0+/−1.6 vs. −11.1+/−1.7, p<0.05). Sinupret-mediated anion secretion in WT MNSE pre-treated with tannic acid was significantly greater than controls; (12.8+/−2.2 vs. 3.6+/−0.9, p<0.05) and still exhibited pronounced CFTR inhibition with INH-172 (−11.5+/−1.7 vs. −9.0+/−1.5, p<0.05) (Figure 2B and 2C). In epithelial monolayers from CFTR^−/−^ mice, pre-incubation with tannic acid abolished the Sinupret-dependent I_SC_ (Figure 2A). These findings suggest that the two major components of ΔI_SC_ stimulated by the formulation are attributable to CFTR-mediated and calcium-activated Cl^−^ secretion.

### Sinupret activates Ca^2+^-mediated, but not cAMP/PKA-dependent cell signaling pathways

**Figure 3:**
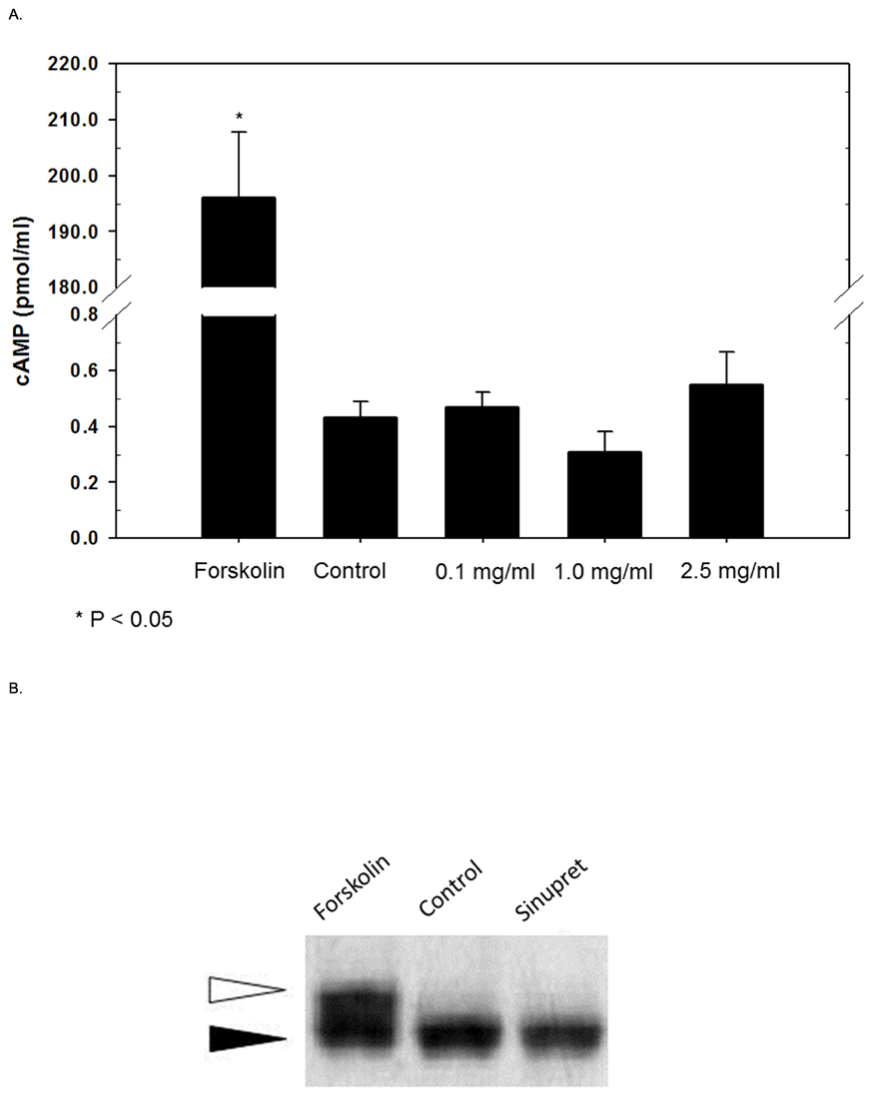
Activation of chloride secretion occurs through a mechanism independent of PKA and R-Domain phosphorylation of CFTR. MNSE cells were exposed to forskolin (20 µM, positive control, P<0.05), vehicle (DMSO), or graded concentrations of Sinupret for 10 minutes prior to assay. Sinupret did not induce a mean increase in cAMP compared to vehicle control, while forskolin led to a marked elevation of cAMP (A). Sinupret does not induce R-domain phosphorylation (B). The recombinant CFTR R-Domain was expressed in stably transfected NIH-3T3 cells and detected by immunoblotting with an antibody to the HA tag (black arrow). A 2–4 kD mobility shift is indicative of R-Domain phosphorylation (white arrow) following 5 minute treatment with forskolin (20 µM, positive control). Sinupret had no detectable effect on R- domain phosphorylation at a maximum concentration of 2.5 mg/ml.

**Figure 4:**
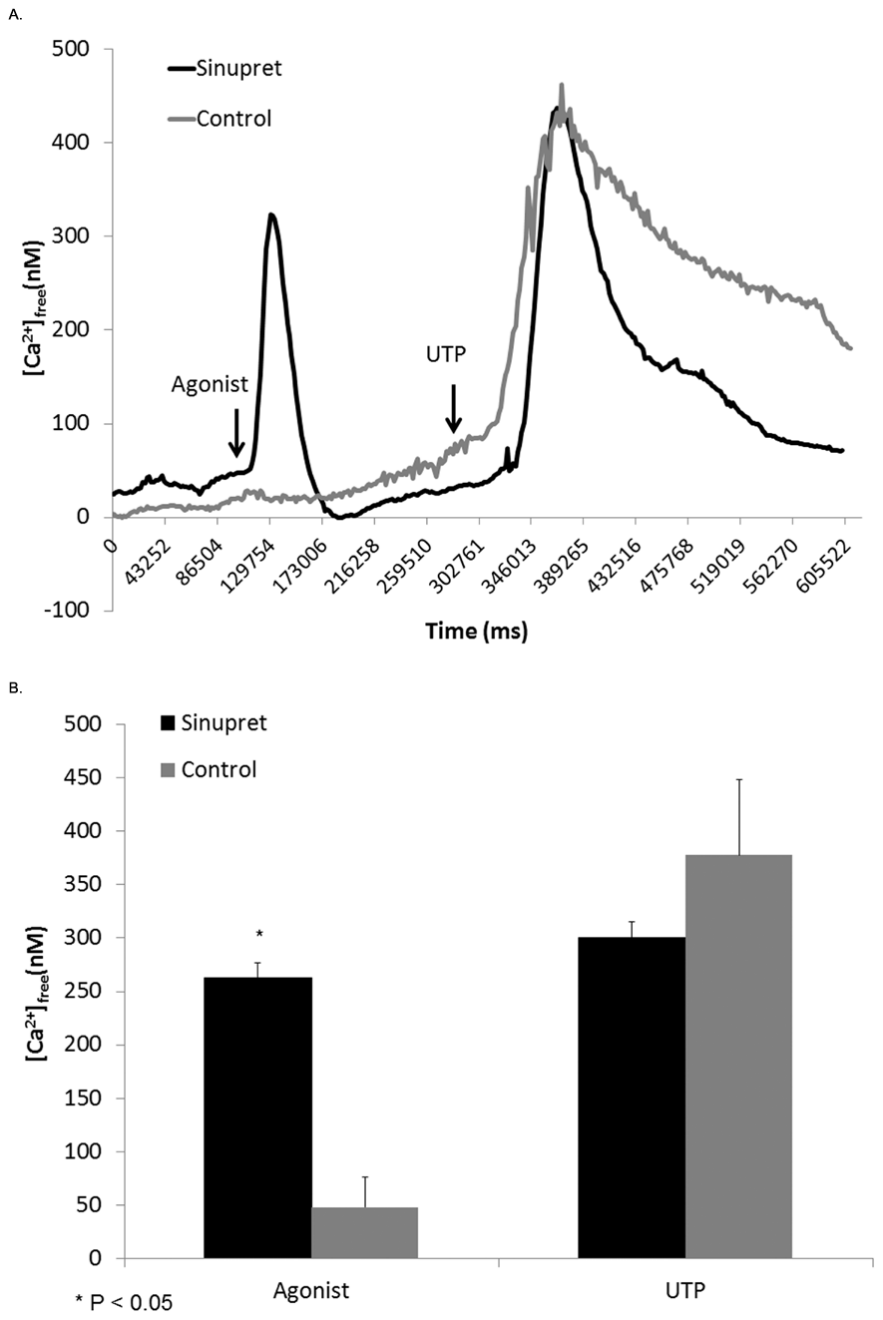
Sinupret raises cytosolic Ca^2+^ concentrations in human sinonasal epithelium. Representative tracings of Fura-2 Ca^2+^ imaging (A). Stimulation of intracellular Ca^2+^ was demonstrated following the addition of Sinupret to HSNE cells. UTP (positive control) also led to increased intracellular Ca^2+^.

CFTR-mediated anion secretion is regulated by cAMP/PKA-dependent second messenger signaling, which results in phosphorylation of the regulatory (R)-domain. TMEM16A-dependent transport relies on fluxes in intracellular Ca^2+^. In order to determine whether Sinupret works via these second messenger pathways, intracellular Ca^2+^, cAMP, and a gel shift assay used to detect R-domain phosphorylation were utilized. Sinupret applied to MNSE cultures at 0.1, 1.0, and 2.5 mg/ml did not increase cAMP (in pmol/ml) in contrast to that seen with the positive control 20 µM forskolin (Figure 3A). Additionally, Sinupret did not confer phosphorylation of the CFTR R-domain (Figure 3B). We performed Fura-2 Ca^2+^ imaging to assess whether the formulation stimulates transport via an increase in [Ca^2+^]_i_ [24]. An increase in [Ca^2+^]_i_ (in nM) was observed by this assay following treatment with 2.5 mg/ml Sinupret (262.8+/−14.4 vs. 48.3+/−28.5, p<0.05). The Sinupret-induced [Ca^2+]^_i_ rise was approximately 70% of the UTP-induced [Ca^2+^]_i_ rise, which served as the positive control (377.8+/−70.3). UTP response following Sinupret (300.7+/−84.1) was not significantly different than UTP alone (Figure 4).

### Sinupret activates TMEM16A and increases CFTR channel open time as judged by whole cell and single channel patch clamp analysis

**Figure 5:**
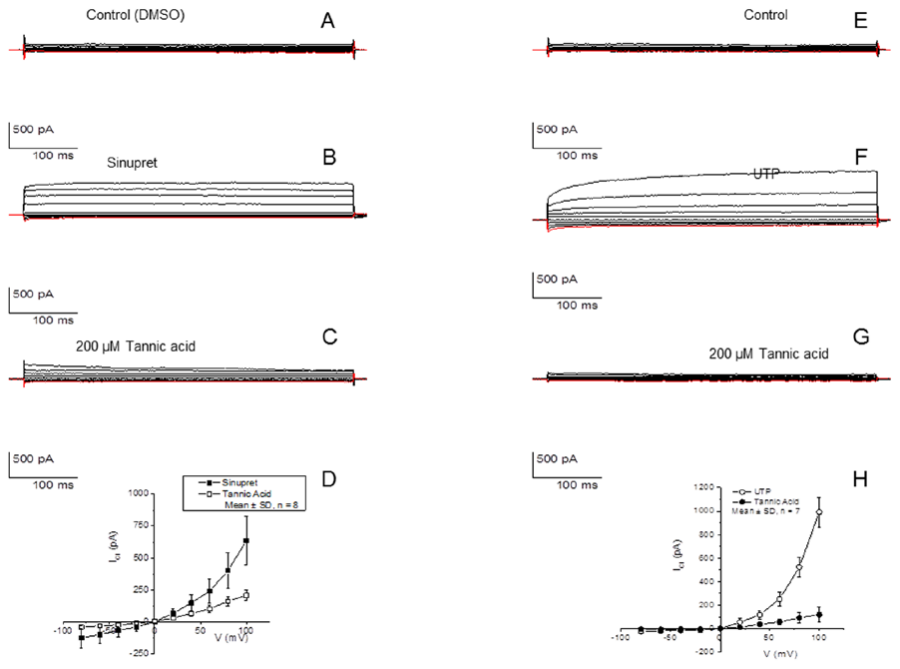
Activation of TMEM16A channels expressed in HEK-293 cells with Sinupret. Control whole cell current was recorded in the presence of DMSO vehicle. DMSO was without any effect on TMEM16A expressed at the surface of HEK cells (A). Perfusion with the solution containing 2.5 mg/ml (B). Sinupret induced the activation of a Cl^−^ current that was inhibited by 200 µM tannic acid (**C**). IV curves are illustrated during the activation of the Cl^−^ current with Sinupret and inhibition by tannic acid when voltage steps were varied from −80 mV to 100 mV in 20 mV increments for 500 ms (D). The cell membrane potential was held at 0 mV throughout the experiment. Control current recorded before cells were treated with UTP - a known activator of TMEM16A channels (E). TMEM16A response to 200 µM UTP in the perfusing solution (F). Inhibition of the UTP activated current with 200 µM tannic acid (G). IV curves (H) showing the activation of the current at a range of voltage stimulation as in (D).

To determine whether TMEM16A was definitively activated by Sinupret, HEK cells stably transfected with TMEM16A were subjected to whole cell patch clamp analysis (Figure 5). Perfusion with Sinupret induced activation of a Cl^−^ current that was inhibited by tannic acid. DMSO controls had no effect on TMEM16A activity at the cell surface, while positive controls had UTP-dependent activation that was prominently blocked by tannic acid.

Application of 2.5 mg/ml Sinupret resulted in activation of a small conductance Cl^−^ channel consistent with CFTR (with the anticipated single channel conductance and INH-172 sensitivity) in 4 out of 14 inside-out patches from CFBE cells. In 4 experiments, ATP/PKA was added to the bath following Sinupret exposure but with no channel activation. For patches in which Sinupret activated CFTR, NPO increased from 0.77±0.26 to 2.21±0.98–a 2.9 fold increase in activity (NPO/n: 0.18±0.06 to 0.43±0.15, where n = number of discernable channel levels). Thus, in the absence of other stimulatory molecules, Sinupret increased CFTR channel Po. This is consistent with our finding of CFTR activation in an intact cell preparation (Figure 1) in which Sinupret increased CFTR channel activity in the absence of *in vitro* stimulation by PKA (Figure 3).

### Sinupret increases CBF *in vitro*

**Figure 6:**
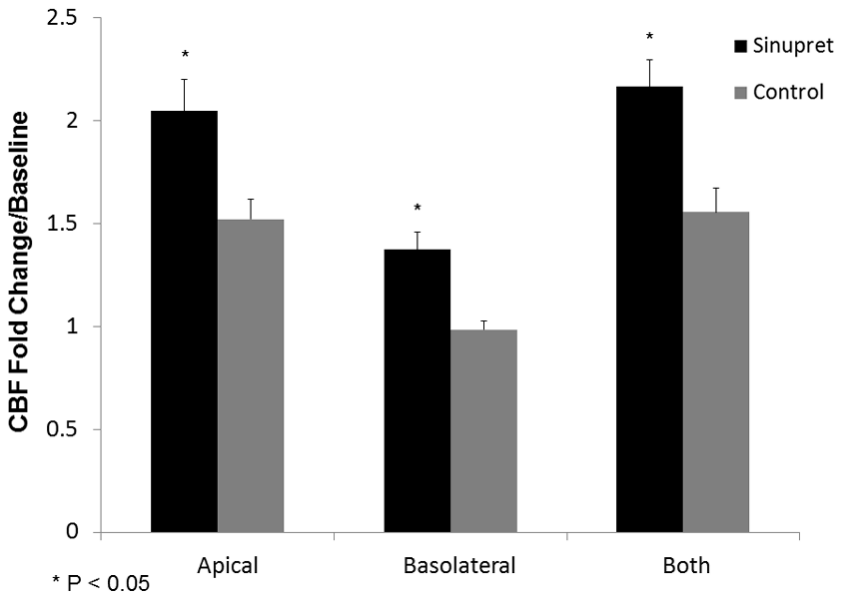
Stimulation of CBF. CBF (fold-change/baseline) was significantly increased when Sinupret was compared to control (vehicle) following apical (2.05+/−0.15 vs. 1.52+/−0.10), basal (1.37+/−0.09 vs. 0.99+/−0.04), or apical + basolateral exposures (2.17+/−0.12 vs. 1.53+/−0.09). (*p<0.05).

CBF is an important component of MCC that responds to elevation of intracellular Ca^2+^. To determine whether Sinupret activates CBF, the drug was applied to the apical, basolateral, or apical+basolateral membranes and compared to corresponding vehicle controls. Sinupret elicited a significant increase in the mean CBF (fold-change/baseline) of MNSE cultures when applied to the apical membrane [2.05+/−0.15 vs. 1.52+/−0.10 (control)], basolateral membrane [1.37+/−0.09 vs. 0.99+/−0.04(control], or both [2.17+/−0.12 vs. 1.53+/−0.09(control)] (p<0.05) (Figure 6).

### Sinupret enhances ASL depth

**Figure 7:**
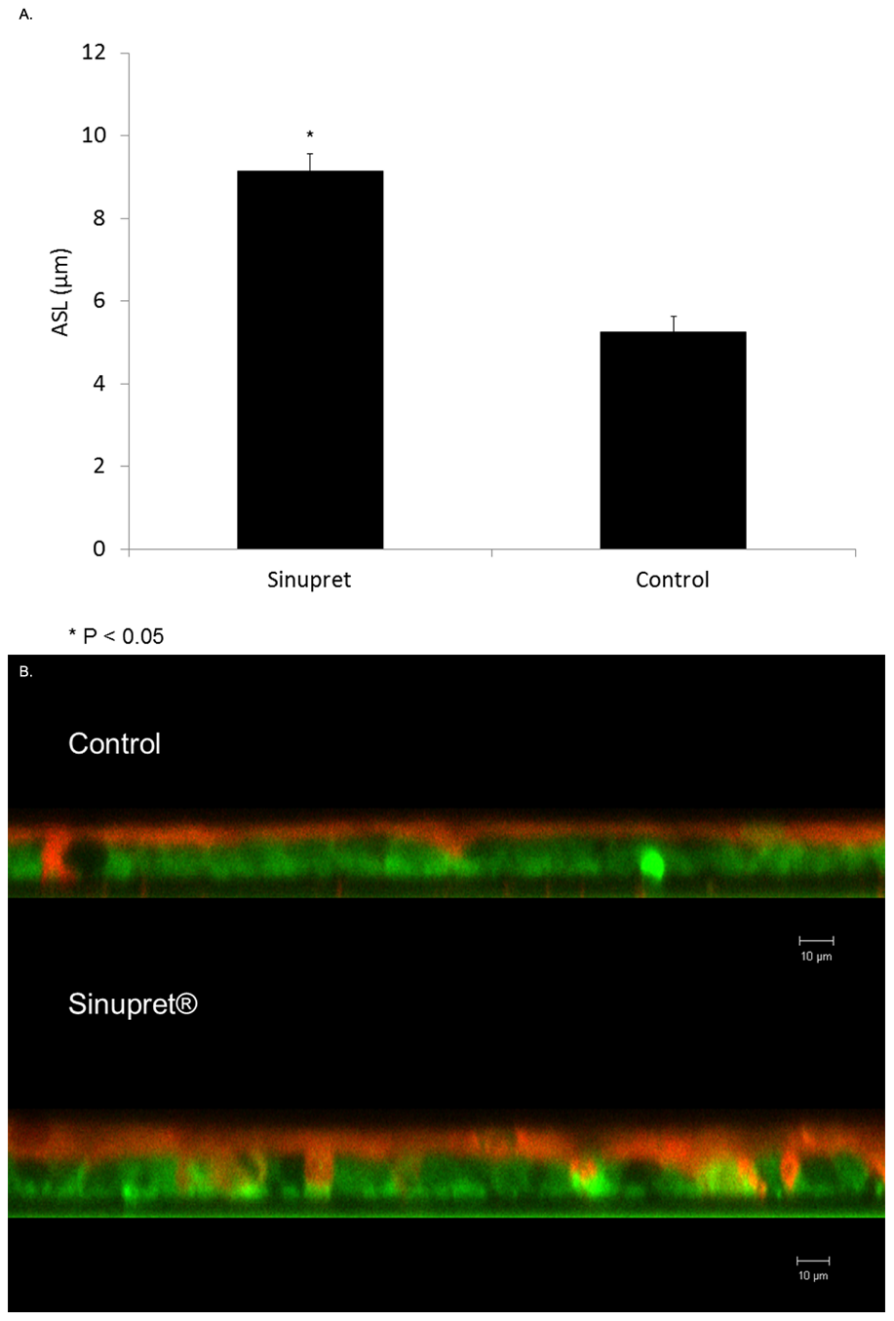
Sinupret hydrates airway surface liquid. Sinupret increased ASL depth (in µm) for murine nasal septal epithelial cultures (9.14±0.42 vs. 5.25±0.38, control, *p<0.05) (A). Confocal images suggest a treatment effect from stimulation of apical Cl^−^ secretion (Red-ASL; Green-Cell marker) (B).

It is well-established that stimulation of transepithelial Cl^−^ secretion leads to airway surface hydration and promotes ASL depth. In order to evaluate effects of the formulation on ASL, MNSE cultures were exposed to Sinupret and stained to visualize overlying surface liquid. Sinupret significantly increased ASL depth in these cultures (in µm: 9.14±0.42 vs. 5.25±0.38, control, *p<0.05) indicating a vigorous treatment effect (Figure 7).

## Discussion

Sinupret has been utilized extensively throughout Europe for more than 70 years as treatment of airway diseases associated with inadequate MCC, and has an excellent safety profile. The medication is registered as a regulated phyto-pharmaceutical in Germany [29], and has been available in the United States for 9 years where it is considered an herbal supplement (not regulated by the Food and Drug Administration) [11]. The drug has been extensively investigated in a variety of scientific studies and clinical settings. Prophylactic administration to mice led to increased resistance against respiratory tract infection following intranasal application of Sendai virus (Parainfluenza viridae) [30]. Mucus clearance properties have been observed in rabbit tracheas *in vivo* [31]. Several clinical trials have tested the effect of Sinupret either alone or in combination with antibiotics for rhinosinusitis. Significant improvement in radiologic outcomes and headache were demonstrated in a randomized, placebo-controlled trial [32]. When used as an adjunctive treatment with antibiotics and/or nasal decongestants in this setting, two clinical studies demonstrated benefit versus placebo [33].

In the present experiments, we establish that Sinupret-stimulates Cl^−^ secretion in part by activating apical CFTR channels. This finding is supported by: 1) a significant decrease in ΔI_SC_ with chemical inhibition (INH-172) in WT airway epithelial monolayers, 2) stimulation of I_SC_ despite pretreatment with tannic acid in WT MNSE, 3) blunted Cl^−^ secretion in CFTR^−/−^ MNSE lacking CFTR, and 4) excised inside-out patch clamp analysis demonstrating increase in CFTR channel Po. Overall Cl^−^ transport remained significantly greater than controls despite absence of CFTR channel function (Figure 1), suggesting either increased Cl^−^ gradient (secretory driving force) due to activation of basolateral K^+^ channels (NKCC1) that promote Cl^−^ transport through TMEM16A, enhanced intracellular Ca^2+^, and/or increased gating of TMEM16A was responsible for the residual transepithelial Cl^−^ secretion in the absence of CFTR. Notably, Cl^−^-dependent I_SC_ was abrogated following addition of the TMEM16A blocker tannic acid in CFTR^−/−^ cell monolayers, indicating that non-CFTR activity was responsible for these findings. It has been reported previously that the absence of CFTR in murine airways may confer enhanced expression or activity of other Cl^−^ secretory pathways, including TMEM16A [34]. Therefore, the Cl^−^ secretory mechanisms available for stimulation in CFTR^−/−^ mice may differ from those in WT. Nevertheless, Fura2-imaging suggests intracellular Ca^2+^ release contributes to TMEM16A activation in this setting, while activation of current in whole cell patch clamp analysis in TMEM16A transfected cells confirms these channels are stimulated by Sinupret. Therefore, Cl^−^ transport is clearly mediated via effects on both apical CFTR and TMEM16A.

Two key components of MCC include ciliary beat and the composition of the ASL. Because Sinupret stimulates intracellular Ca^2+^ signaling cascades known to stimulate both TMEM16A-mediated Cl^−^ transport and CBF in mammalian respiratory epithelial cells [16], [35], it is not surprising that an increase in CBF as well as ASL depth was noted in this study. Stimulation of CFTR-dependent pathways through increased channel Po (as opposed to cAMP/PKA-mediated phosphorylation of the R-domain) may also contribute [36], [37] and are likely related to the flavonoid components of the formulation [4], [6]. Activation of mechanisms such as these would improve MCC and probably account for underlying health benefits of the drug.

## Conclusions

Sinupret likely derives its clinical benefit, at least in part, through stimulating transepithelial Cl^−^ secretion, CBF, and MCC. Augmenting fluid and electrolyte secretion represents a means of improving mucus clearance in individuals affected by acute or chronic rhinosinusitis, specifically in the setting of WT CFTR. The present studies also point to molecular pathways that might be targeted as a means to improve therapy of MCC in diseases such as CRS and COPD in the future.
